# Meta-analysis of yield response of foliar fungicide-treated hybrid corn in the United States and Ontario, Canada

**DOI:** 10.1371/journal.pone.0217510

**Published:** 2019-06-05

**Authors:** Kiersten A. Wise, Damon Smith, Anna Freije, Daren S. Mueller, Yuba Kandel, Tom Allen, Carl A. Bradley, Emmanuel Byamukama, Martin Chilvers, Travis Faske, Andrew Friskop, Clayton Hollier, Tamra A. Jackson-Ziems, Heather Kelly, Bob Kemerait, Paul Price, Alison Robertson, Albert Tenuta

**Affiliations:** 1 Department of Plant Pathology, University of Kentucky Research and Education Center, Princeton, Kentucky, United States of America; 2 Department of Plant Pathology, University of Wisconsin-Madison, Madison, Wisconsin, United States of America; 3 Department of Botany and Plant Pathology, Purdue University, West Lafayette, Indiana, United States of America; 4 Department of Plant Pathology and Microbiology, Iowa State University, Ames, Iowa, United States of America; 5 Department of Biochemistry, Molecular Biology, Entomology and Plant Pathology, Delta Research and Extension Center, Mississippi State University, Stoneville, Mississippi, United States of America; 6 Department of Agronomy, Horticulture, and Plant Science, South Dakota State University, Brookings, South Dakota, United States of America; 7 Department of Plant, Soil and Microbial Sciences, Michigan State University, East Lansing, Michigan, United States of America; 8 Department of Plant Pathology, Division of Agriculture, Lonoke Extension Center, Lonoke, United States of America; 9 Department of Plant Pathology, North Dakota State University, Fargo, North Dakota, United States of America; 10 Department of Plant Pathology and Crop Physiology, LSU AgCenter, Baton Rouge, Louisiana, United States of America; 11 Department of Plant Pathology, University of Nebraska-Lincoln, Lincoln, Nebraska, United States of America; 12 Department of Entomology and Plant Pathology, University of Tennessee West Tennessee Research and Education Center, Jackson, Tennessee, United States of America; 13 Department of Plant Pathology, University of Georgia, Tifton, Georgia, United States of America; 14 Ontario Ministry of Agriculture, Food, and Rural Affairs, University of Guelph-Ridgetown, Ridgetown, Ontario, Canada; College of Agricultural Sciences, UNITED STATES

## Abstract

**Background:**

Foliar fungicide applications to corn (*Zea mays* L.) occur at one or more application timings ranging from early vegetative growth stages to mid-reproductive stages. Previous studies indicated that fungicide applications are profitable under high disease pressure when applied during the tasseling to silking growth stages. Few comprehensive studies in corn have examined the impact of fungicide applications at an early vegetative growth stage (V6) compared to late application timings (VT) for yield response and return on fungicide investment (ROI) across multiple locations.

**Objective:**

Compare yield response of fungicide application timing across multiple fungicide classes and calculate the probability of positive ROI.

**Methods:**

Data were collected specifically for this analysis using a uniform protocol conducted in 13 states in the United States and one province in Canada from 2014–2015. Data were subjected to a primary mixed-model analysis of variance. Subsequent univariate meta-analyses, with and without moderator variables, were performed using standard meta-analytic procedures. Follow-up power and prediction analyses were performed to aid interpretation and development of management recommendations.

**Results:**

Fungicide application resulted in a range of yield responses from -2,683.0 to 3,230.9 kg/ha relative to the non-treated control, with 68.2% of these responses being positive. Evidence suggests that all three moderator variables tested (application timing, fungicide class, and disease base level), had some effect (α = 0.05) on the absolute difference in yield between fungicide treated and non-treated plots ($\bar{D}$). Application timing influenced $\bar{D}$, with V6 + VT and the VT application timings resulting in greater yield responses than the V6 application timing alone. Fungicide formulations that combined demethylation inhibitor and quinone outside inhibitor fungicides significantly increased yield response.

**Conclusion:**

Foliar fungicide applications can increase corn grain yield. To ensure the likelihood of a positive ROI, farmers should focus on applications at VT and use fungicides that include a mix of demethylation inhibitor and quinone outside inhibitor active ingredients.

## Introduction

Foliar fungicide applications to hybrid corn (*Zea mays* L.) have increased since the mid-2000s, due to reports that fungicides provide physiological benefits to crop plants that enhance yield even in the absence of disease [1–4]. Foliar fungicide applications in corn have been promoted at one or more timings ranging from early vegetative to late reproductive growth stages. The primary purpose of early vegetative stage (three-leaf collar to eight leaf collar growth stages; V3-V8; [5]) applications is to gain yield advantages from physiological benefits [6], while fungicide applications at the tasseling-silking corn growth stage (VT-R1) target both foliar disease management and yield gain from physiological response to fungicide [7]. Previous studies have indicated applications occurring at VT-R1 are most likely to be profitable when conditions favor disease development, such as planting hybrids susceptible to foliar diseases like gray leaf spot (caused by *Cercospora zeae-maydis*), northern corn leaf blight (caused by *Exserohilum turcicum*) and southern rust (caused by *Puccinia polysora*), planting into fields with high levels of corn residue, irrigated fields, and/or fields under continuous corn production [3, 7].

Despite research that indicates foliar fungicide applications at the VT-R1 timing are most likely to provide an economic response in corn, the authors observed that applications occurring during the early vegetative growth stages are still marketed to farmers as a way to improve yield. This is because farmers can apply foliar fungicides to corn prior to tasseling using ground-driven spray equipment. Moreover, other additives may be included in the application, such as postemergence herbicides and foliar fertilizers, minimizing the number of times a field is sprayed, which can reduce fuel costs. The overall application cost of applying fungicide with ground equipment at an early vegetative stage may be less than an application occurring at tasseling or later, because tasseling or post-tasseling applications are typically applied with aerial equipment, such as helicopters or planes [8].

Previous studies examining the impact of early vegetative applications on corn compared to tasseling applications have indicated that early vegetative applications did not have greater yield compared to a non-treated control in single location trials [9–14]. To date, few trials have compared the effect of foliar fungicide application timing on disease management and yield across multiple locations. One such trial, using two locations and four location-years, resulted in similar findings as individual location trials, where vegetative growth stage applications occurring from the five leaf-collar to eight leaf-collar stages (V5-V8) did not have higher yield compared to applications occurring at the early reproductive growth stages of tasseling or blister (VT to R2; [15]). To our knowledge, no comprehensive studies have examined the impact of early vegetative fungicide applications of multiple fungicide classes in corn compared to later timings across different environments and production practices or examined the return on investment of fungicide application by fungicide timing. These comprehensive analyses aid in drawing meaningful conclusions about the impact of foliar fungicide timing on yield and profitability in corn.

Due to the complexity of analyzing data from numerous trials across locations that might differ in experimental design, meta-analysis has emerged as a useful tool in phytopathology to analyze large, multi-site year datasets [6, 7, 16, 17]. Originally developed for the social sciences, meta-analysis has become important for phytopathologists who deal with large, complex, multi-site analyses, especially when determining fungicide efficacy across numerous environments [18, 19]. The meta-analysis presented here examines results from original trials conducted across 13 states and one province in the United States (US) and Canada, respectively, over two years with a uniform trial protocol consisting of 19 treatments. The objective of this analysis was to estimate mean impacts of fungicide application timing and class on corn yield and return on fungicide application investment using data collected from retailers on current product and application costs.

## Materials and methods

### Data set

In 2014 and 2015, members of the Corn Disease Working Group (CDWG) were invited to participate in a uniform trial protocol to assess efficacy of fungicide applications on foliar disease and yield. The protocol consisted of foliar fungicides applied at the six collar vegetative growth stage of corn (V6), the tasseling growth stage (VT), or an application at V6 followed by an application at VT (V6 + VT). Members of the CDWG are plant pathologists within the US and Canada who meet annually to discuss research and extension needs related to corn diseases. Participation in conducting the trials was voluntary. Treatments were selected based on what the CDWG perceived to be the most commonly promoted fungicide active ingredients for each timing (Table 1).

**Table 1:** Fungicide product information and application timing for uniform fungicide application timing trials conducted in 2014 and 2015 in the United States and Ontario, Canada.

Fungicide active ingredient	Fungicide class(es)	Fungicide resistance action committee fungicide code	Fungicide rate (l/ha)	Fungicide application timing
**Pyraclostrobin + fluxapyroxad**	QoI^a^ + SDHI^b^	11 + 7	0.088	V6^d^
**Azoxystrobin**	QoI	11	0.177	V6
**Prothioconazole + trifloxystrobin**	QoI + DMI^c^	11 + 3	0.059	V6
**Picoxystrobin**	QoI	11	0.088	V6
**Fluoxastrobin + flutriafol**	QoI+ DMI	11+3	0.148	V6
**Metconazole + pyraclostrobin**	QoI + DMI	11 + 3	0.295	VT^e^
**Prothioconazole + trifloxystrobin**	QoI + DMI	11 + 3	0.118	VT
**Azoxystrobin + propiconazole**	QoI + DMI	11 + 3	0.310	VT
**Picoxystrobin**	QoI	11	0.177	VT
**Fluoxastrobin + flutriafol**	QoI + DMI	11 + 3	0.148	VT
**Cyproconazole + picoxystrobin**	QoI + DMI	11 + 3	0.201	VT
**Propiconazole**	DMI	3	0.118	VT
**Tetraconazole**	DMI	3	0.118	VT
**Fluxopyroxad + pyraclostrobin fb**^**f**^ **metconazole + pyraclostrobin**	SDHI + QoI fb DMI + QoI	11 + 3	0.088 fb 0.295	V6 fb VT
**Azoxystrobin fb azoxystrobin + propiconazole**	QoI fb QoI + DMI	11 + 3	0.177 fb 0.310	V6 fb VT
**Prothioconazole + trifloxystrobin fb prothioconazole + trifloxystrobin**	QoI + DMI fb QoI + DMI	11 + 3	0.118 fb 0.118	V6 fb VT
**Picoxystrobin fb cyproconazole + picoxystrobin**	QoI fb DMI + QoI	11 + 3	0.177 fb 0.201	V6 fb VT
**Fluoxastrobin + flutriafol fb fluoxastrobin + flutriafol**	QoI + DMI fb QoI + DMI	11 + 3	0.148 fb 0.148	V6 fb VT

^a^QoI = Quinone outside inhibiting fungicide class

^b^SDHI = Succinate dehydrogenase inhibiting fungicide class

^c^DMI = Demethylation inhibiting fungicide class

^d^V6 = Six leaf collar growth stage corn

^e^VT = Tasseling growth stage of corn

^f^fb = followed by

All trials were conducted by the co-authors of this article at the respective university research farms or on-farm locations in the US and Ontario, Canada (Table 2). All trials were conducted with treatments arranged in a randomized complete block design in a single trial. Treatments were replicated at least four times in each trial and included at least three of the fungicide treatments included on the uniform protocol and a non-fungicide treated control. Hybrids adapted to each location were used and considered at least moderately susceptible to prevalent foliar diseases in each region, which included gray leaf spot, northern corn leaf blight, and southern rust. In all experiments, except Ontario in 2014, a single hybrid was used. In Ontario in 2014, the experiment was conducted using two hybrids; each hybrid was considered an individual trial. Hybrid choice was left up to the discretion of the pathologist in each location and was not considered further in the analyses.

**Table 2:** Location information for uniform fungicide application timing trials conducted in 2014 and 2015 in the United States and Ontario, Canada.

State/province	Experimental locations	Years trial conducted
**Arkansas**	Altheimer	2014, 2015
**Georgia**	Attapulgus	2014, 2015
**Illinois**	Auburn, DeKalb, Dixon Springs, Monmouth, Urbana	2014
**Indiana**	Vincennes, West Lafayette	2014, 2015
**Iowa**	Boone, Kanawha, Lewis, Nashua, Sutherland	2014, 2015
**Louisiana**	Baton Rouge, Winnsboro	2014, 2015
**Michigan**	East Lansing	2015
**Mississippi**	Stoneville	2015
**Nebraska**	Clay Center	2015
**North Dakota**	Fargo, Davenport	2014, 2015
**Ontario, Canada**	Ridgetown	2014, 2015
**South Dakota**	Beresford	2015
**Tennessee**	Milan	2015
**Wisconsin**	Arlington	2014, 2015

Experimental plot size varied across trial locations, but each plot had at least two rows planted at 0.76-m spacing and was at least 6.1 m long. All disease and yield data were collected from the two center rows. Experiments followed local recommendations for general crop management including fertility and weed management. All treatments were ground-applied using self-propelled high-clearance sprayers, or hand-held booms at 56 to 75 l/ha. No adjuvants were included in fungicide applications except in Iowa in 2015, where non-ionic surfactant was used. Disease severity was assessed as percent severity of each disease present on the ear leaf of at least 5 plants per plot at the late dough-early dent growth stages (R4-R5). Prior to analysis, disease severity for each disease was combined into total percent severity for all diseases present on the ear leaf. Only trials including disease severity data were included in the analysis. Grain was harvested in each trial with small-plot combines, and yields were calculated and standardized to 15.5% moisture prior to analysis.

### Quantitative data synthesis

#### Analysis of variance for individual trials

Original data were collected from each participating state/province, and mixed-model analysis of variance (ANOVA) was performed using PROC GLIMMIX in SAS version 9.4 (SAS Institute Inc., Cary, NC). Fungicide treatment was considered a fixed effect, and replication was considered a random effect in the ANOVA model. A normal distribution was used for all analyses. Treatment least-square means and residual variances for each trial were obtained from the primary ANOVA. In states where multiple trials were conducted in each year, each trial was analyzed individually and considered as an independent study for the meta-analysis (S1 Fig). The denominator degree of freedom for the test of fixed effects was determined by Kenward-Roger approximation defined as the ddfm = kr option in the model statement. Treatment lsmeans were obtained using the *lsmeans* statement.

#### Effect size and meta-analysis of the treatment effect

The absolute yield difference (*D*) between the fungicide treated and non-treated control was used as the effect size. Computation of *D* was performed by subtracting the non-treated control mean yield (${\bar{X}}_{control}$) from the treatment yield (${\bar{X}}_{treatment}$) such that *D* = ${\bar{X}}_{treatment}‑{\bar{X}}_{control}$. The difference in sampling variance was computed as *Si*^2^ = (2×*V*)/*n*, where *i*, represents the *i*th study, *V* represents the residual variance, which was obtained from primary ANOVA, and *n* represents the replication of the trial. Univariate random-effect meta-analysis was performed to estimate the overall ($\bar{D}$) and among study variance (${\hat{\sigma }}^{2}$) using PROC MIXED in SAS, where trial was defined as random effect factor. Weight for each study was given as the inverse of sampling variance, weight = (1/*Si*^2^) [20]. The confidence interval of the mean was estimated at 95% using the *cl* option in the model statement. Percent yield increase was calculated as ($D/{\bar{X}}_{control}$) x 100.

#### Study heterogeneity and moderator variables

Significance of study heterogeneity (among-study variance), was tested using a likelihood ratio statistic as described previously [19]. Given that the study heterogeneity was significantly different from zero, categorical moderator variables of fungicide application timing, fungicide class as defined by the Fungicide Resistance Action Committee (FRAC; [20]), and baseline disease level (disease base) were tested. A mixed effect model used moderators as fixed effect factors to determine whether, and how much, the moderator variable explained the heterogeneity in the estimates.

To use disease base as a moderator variable, trials were grouped into two categories based on percent disease severity in non-treated control plots as low disease (< 5%) and high disease (≥ 5%). A 5% cutoff was used since Paul et al. [7] demonstrated that this cutoff was useful in explaining the significance of success in using fungicide for management of gray leaf spot. For fungicide class, trials were re-grouped into five categories based on FRAC classes of the fungicides used in the trials, and a separate analysis performed (Table 1). Fungicides were applied at V6, VT, or V6 + VT; therefore, trials were divided again into these three categories to use fungicide application as a moderator variable. Furthermore, fungicide classes were analyzed separately (if used multiple times) using time of application and fungicide product as moderator variables to determine how application timing and fungicide product within the fungicide class affected the response. The number of trials used in the analysis from each category is given in Table 3. The percent variability explained by each moderator variable was computed as follows; {(*v*–*r*)/*v*}, × 100, where v is the among study variances before moderator variables are specified and r is the among study variances after moderator variables are specified.

**Table 3:** Effect of moderator variables on yield response to fungicide, with the corresponding statistics based on mixed-effect meta-analysis for trials performed at 13 US states and Ontario, Canada in 2014 and 2015.

						Effect size^e^					
Moderator variables^a^	Category^b^	K^c^	Mean yield NTC (kg/ha)^d^	$\bar{D}$	$se(\bar{D})$	*CI*_*L*_	*CI*_*U*_	*t*	*P*	*PW*	Yield increase (%)
**Application timing**	V6 + VT	122	12,146	493.9	51.7	392.5	595.3	9.55	< .0001	1.0	4.1
**(18%, *P* <0.01)**	V6	125	12,205	127.4	51.3	26.5	227.6	2.48	0.0133	0.7	1.0
	VT	189	11,982	376.8	42.5	293.5	460.1	8.87	< .0001	0.9	3.1
**Disease base**	Low	187	11,557	410.8	46.6	319.4	502.2	8.81	< .0001	0.9	3.5
**(4%, *P* = 0.04)**	High	249	12,493	286.4	36.6	214.6	358.1	7.82	< .0001	0.9	2.3
**Fungicide class**	DMI	20	11,556	155.7	139.0	-116.8	428.2	1.12	0.2627	0.2	1.3
**(11%, *P* < 0.01)**	QoI	86	12,084	180.5	64.1	54.8	306.2	2.82	0.0049	0.8	1.5
	DMI + QoI	272	12,098	390.8	35.6	321.0	460.5	11.0	< .0001	1.0	3.2
	SDHI + QoI	29	12,257	139.6	107.8	-71.6	350.8	1.30	0.1951	0.2	1.1
	DMI + SDHI + QoI	29	12,257	574.4	107.8	363.2	785.6	5.33	< .0001	0.9	4.7

^a^ Number with percentage in parenthesis is the percentage of the study heterogeneity explained by the moderator variable and *P* value is test of the null hypothesis of categories within each moderator variable are not statistically different. The variability percentage explained by each moderator variable was computed as follows; {(*v*–*r*)/*v*}, × 100, where v and r are the among study variances before and after the moderator variables are specified, respectively.

^b^ V6 = sixth leaf collar and VT = tasseling growth stages of corn. For the disease base, low is < 5% disease severity, high is ≥ 5% disease severity. For fungicide class, trials were grouped in to five categories based on Fungicide Resistance Action Committee classes of the fungicides: DMI = demethylation inhibitors, QoI = quinone outside inhibitors, and SDHI = succinate dehydrogenase inhibitors.

^c^ K = number of trials used in the analysis.

^d^ Mean yield of non-treated control plots (NTC) in kilograms per hectare (kg/ha).

^e^ $\bar{D}$ = Mean yield difference between fungicide treated and NTC, $\mathrm{se}(\bar{D})$ = standard error of the difference, *CI_L_* = lower limits *CI_U_* = upper limits of the 95% confidence interval of the $\bar{D}$, *P* is the probability of rejecting null hypothesis that the effect size is not different from zero. Percent yield increase was calculated as ($\bar{D}/{\bar{X}}_{control}$) x 100, *PW* is the two-sided power analysis where H_0_: $\bar{D}$ = 0; α = 0.05; *df* = *K*-1 for K observations.

#### Power analysis

A post-hoc power analysis was performed for each fungicide class, application timing, and disease base within each analysis, where the null hypothesis was *H_0_: ζ* = 0 [18]. Student’s t-statistic ($t=\hat{\zeta }/SE(\hat{\zeta })$ was calculated, and the two-sided test of power was estimated by *Power = 1-F_f_* (F^*^_0.95,1,df_;1,df,ϕ^*2*^). In this null hypothesis statement, *ζ* = the effect size of the *K*th study for a fungicide class, application timing, or disease base. Following the convention of Madden and Paul [19], the power analysis enables the user to determine if the study was underpowered, that is, the lack of significant effect could be due to small sample size. Given that the number of observations might be considered low for some treatment combinations, the power analysis was used to guide subsequent interpretation of the results. Power analysis was performed with the aid of the metapower macro [Madden, *unpublished*] with K—1 *df*. Power estimates less than 0.8 were considered low power, indicating that more studies might be needed to find significant differences given $\hat{\zeta }\;and\;SE(\hat{\zeta }).$

### Prediction and risk analysis

The probability of recovering the investment on the fungicide program was also determined based on the effect size ($\bar{D}$) and between study heterogeneity (${\hat{\sigma }}^{2}$) estimated from the meta-analyses [6]. Fungicide class and timing were significant (*P*<0.05), thus, the probability was estimated for QoI and DMI + QoI fungicide classes and timings that were significantly different from each other (Table 4) using a range of application costs and corn market prices. To determine the average costs of fungicide application and subsequent return on investment from fungicide timing and class, co-authors requested specific quotes for prices of the treatment products from farm chemical retailers, agribusiness entities, and others to determine the average cost of fungicide application in the United States and Ontario, Canada. Costs of ground vs. aerial fungicide application were also solicited and compiled to calculate mean and median total fungicide application cost per fungicide product tested (product + application).

**Table 4:** Influence of application timing on yield response to fungicide from quinone outside inhibitors (QoI), and a premix of demethylation inhibitors (DMI) and QoI fungicide classes with the corresponding statistics based on mixed-effect meta-analysis of trials conducted in 12 U.S. states and Ontario, Canada in 2014 and 2015.

					Effect size^d^						
Fungicide class	Application timing^a^	K^b^	Mean yield NTC (kg/ha)^c^	$\bar{D}$	$\mathrm{se}(\bar{D})$	*CI*_*L*_	*CI*_*U*_	*t*	*P*	*PW*	Yield increase (%)
**QoI**	V6 + VT	20	12,040	452.8	101.5	254.1	651.9	4.46	< .0001	0.9	3.8
**(*P* <0.01)**	V6	38	12,086	52.3	74.8	-94.4	199.0	0.70	0.4845	0.1	0.4
	VT	28	12,114	222.8	89.6	47.1	398.4	2.49	0.0129	0.7	1.8
**DMI + QoI**	V6 + VT	73	12,130	480.8	69.8	344.0	617.6	6.89	< .0001	1.0	4.0
**(*P* <0.01)**	V6	58	12,257	172.4	77.8	19.9	324.9	2.22	0.0267	0.6	1.4
	VT	141	12,016	432.1	50.8	332.4	531.8	8.50	< .0001	1.0	3.6

^a^ V6 = sixth leaf collar and VT = tasseling growth stages of corn.

^b^ K = number of trials used in the analysis.

^c^ Mean yield of non-treated control plots (NTC) in kilograms per hectare (kg/ha).

^d^ $\bar{D}$ = Mean yield difference between fungicide treated and NTC, $\mathrm{se}(\bar{D})$ = standard error of the difference, *CI_L_* = lower limits *CI_U_* = upper limits of the 95% confidence interval of the difference, *P* is the probability of rejecting null hypothesis that the effect size is not different from zero. Percent yield increase was calculated as ($\bar{D}/{\bar{X}}_{control}$) x 100, *PW* is the two-sided power analysis where H_0_: $\bar{D}$ = 0; α = 0.05; *df* = *K*-1 for K observations

The probability estimates were computed as *p* = $\left\{\phi [(\bar{D}-C/)/\hat{\sigma }]\right\}$ × 100; where ϕ = the cumulative standard normal function, *C* (constant) = an estimated corn yield that equals the fungicide costs $\bar{D}$ = the effect size, and $\hat{\sigma }$ = the among-study standard deviation [7, 18].

## Results

**Fig 1:**
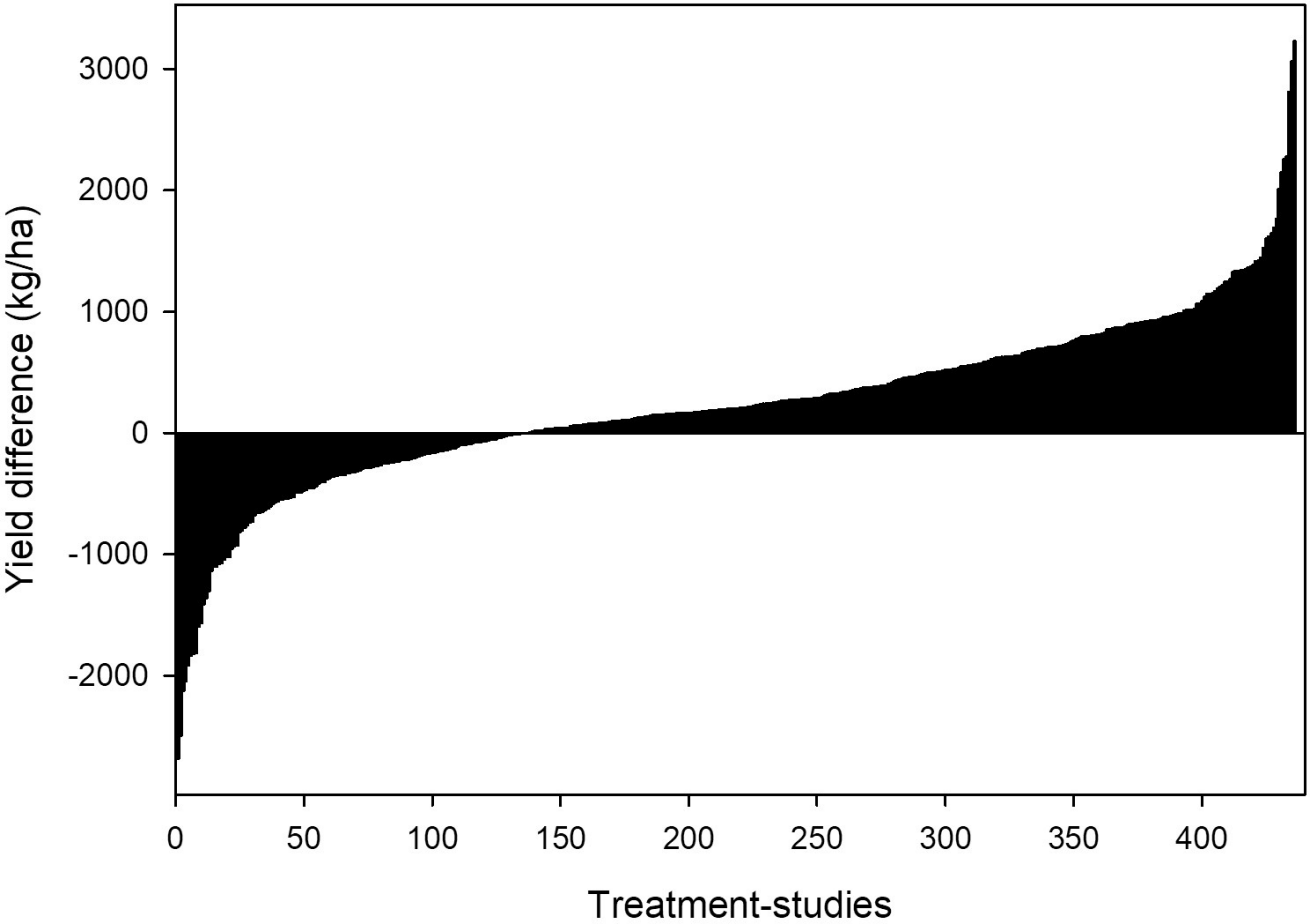
Distribution of mean yield difference (diff.) in kilogram per hectare (kg/ha) between the fungicide treatments and non-treated controls across trials conducted in 13 US states and Ontario, Canada during 2014 and 2015. Each bar represents the difference between the fungicide treatment and non-treated control averaged over four to six replications (K = 436).

Yield response to fungicide application across all trials ranged from -2,683.0 to 3,230.9 kg/ha relative to the non-treated control (Fig 1). Of the 436 treatment-studies, 68.2% had a positive yield response, meaning regardless of application timing, fungicide active ingredient, or disease-base, greater yields occurred in fungicide treated plots than non-treated control plots. The overall yield response to fungicide application was 332.9 ± 29.1 kg/ha (95% CI = 275.8–389.8 kg/ha) and was significantly different from zero (*P* < 0.001).

The among study variance (${\hat{\sigma }}^{2}$ = 157,790) was statistically different from zero (*P* < 0.001) providing evidence that there was considerable variation in yield response among trials. This was partly explained by the addition of separate analyses using moderator variables. Among the three moderator variables used (application timing, fungicide class, and disease base level), all had a significant effect on $\bar{D}$ providing evidence that these moderator variables were suitable in explaining heterogeneity (Table 3). The moderator test of application timing influenced $\bar{D}$, with statistically similar (*P* = 0.08) V6 + VT and VT application timings resulting in greater yield responses than the V6 timing. Base disease level also affected the $\bar{D}$ with greater yield responses observed in trials with low disease severity (< 5%) across all application timings (*P* = 0.04; Table 3). Furthermore, only 4% of the study heterogeneity was explained by disease base level. Fungicide class also influenced $\bar{D}$. The greatest yield response was observed in treatments with DMI + SDHI + QoI fungicide classes (574.4 kg/ha), which was followed by DMI + QoI (390.8 kg/ha), and QoI (180.5 kg/ha) fungicide classes. Treatments with DMI alone or SDHI + QoI fungicide classes did not result in substantial yield responses (Table 3).

Within the fungicide classes of QoI and DMI + QoI, fungicide timing resulted in a significant $\bar{D}$ (Table 4). For the QoI fungicide class, VT and V6 + VT application, evidence suggests that the mean yield response with a VT application was similar to the yield response of a V6 + VT application (*P* = 0.09; Table 4). The same was true for DMI + QoI fungicides, with even less evidence for any difference in yield response for applications at VT vs. V6 + VT (*P* = 0.57). Applications of QoI fungicides at V6 resulted in a marginal yield response with a $\bar{D}$ of just 52.3kg/ha. For DMI + QoI fungicides at V6, evidence was stronger for a yield response (*P* = 0.0267; Table 4). Parsing the dataset further, analysis of fungicide active ingredient within each fungicide class resulted in little explanation of study heterogeneity for QoI (*P* = 0.5081) and DMI + QoI fungicides (*P* = 0.7314). Thus, application of QoI or QoI + DMI was optimal (maximizing yield while reducing number of applications) at the VT application timing (Table 4).

### Prediction and risk analysis

Probability of recovering the fungicide cost is presented for QoI and DMI + QoI fungicides classes only, since these were the fungicide classes most typically promoted for use at V6, and VT applications at the time this research was conducted. Furthermore, evidence for economically meaningful effect sizes was strong (*P* < 0.05) for the moderator effect of application timing for both fungicide classes. For risk analysis, focus was placed on a single application occurring at growth stage V6 and a single application occurring at growth stage VT for both fungicide classes. Fungicide application timing resulted in weak differences (*P* > 0.05) between VT and V6 +VT application timings. The V6 + VT program requires greater input costs to implement compared to the VT application program (two applications vs. one application).

**Fig 2:**
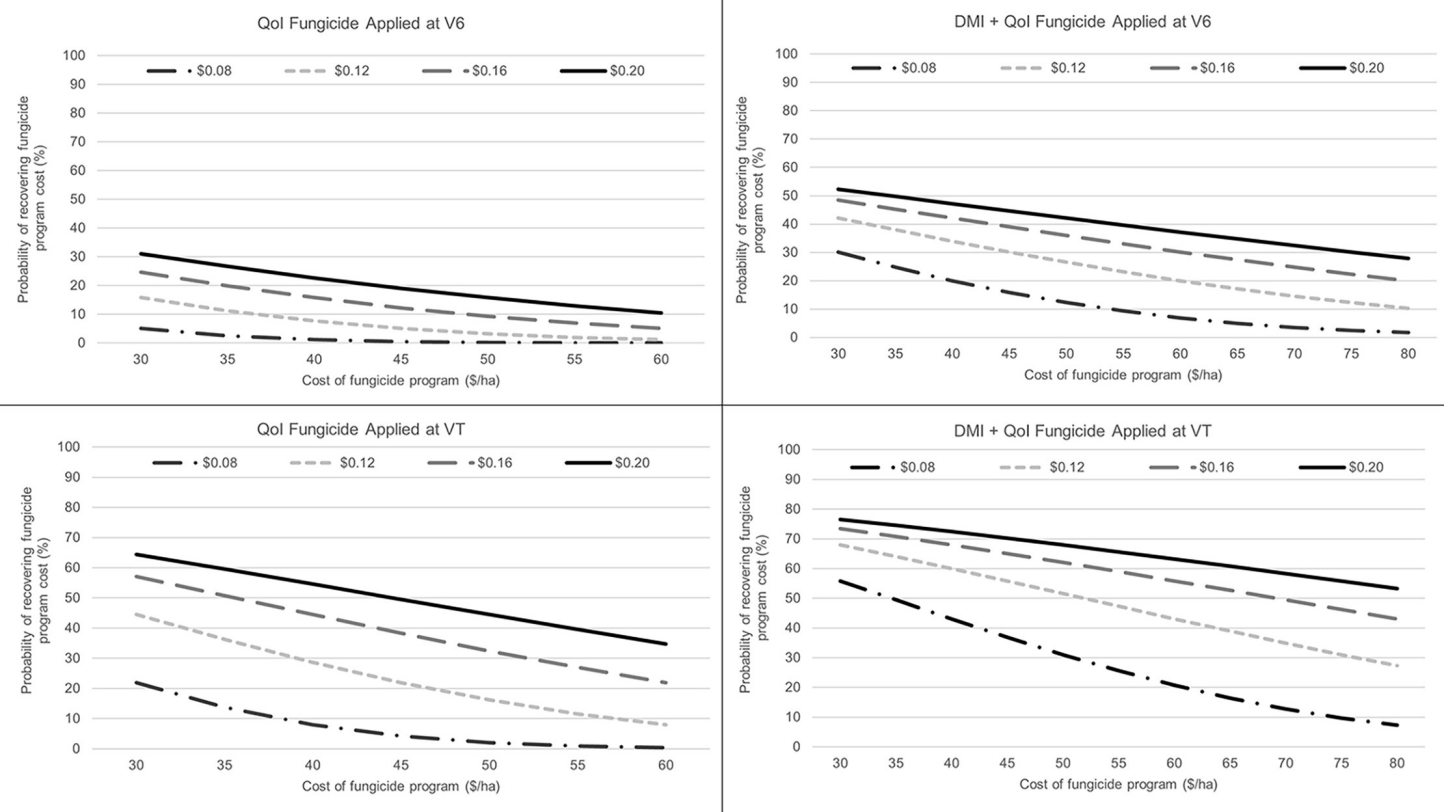
Probability of recovering fungicide cost for a range of corn market prices in $/kg, and fungicide costs estimated for two fungicide classes, quinone outside inhibitors (QoI) and demethylation inhibitors (DMI) + QoI, applied at V6 (six leaf collar growth stage) or VT (tasseling growth stage of corn), based on estimated yield differences and between-study variances from meta-analyses.

A range of fungicide program costs (based on current fungicide retail price and application cost) and corn prices were used to calculate probability of a break-even return on investment (ROI; designated as P*_gain_*) for each fungicide class for the V6 and VT application timings (Table 5). For the QoI fungicide class, program cost ranged from $30 to $55/ha for a single application. In all cases the probability of ROI increased with increasing corn price at a given fungicide cost and decreased with increasing fungicide cost at a given corn price (Fig 2). For example, the P*_gain_* decreased from 25 to 9% when fungicide cost increased from $30 to $55/ha given a corn price of $0.16/kg, and increased from 5 to 31% when corn price increased from $0.08 to $0.20/kg with the fungicide program cost held constant at $30/ha, for a QoI fungicide applied only at V6. The P*_gain_* was less than 31% in all price-cost combinations for QoI at the V6 application timing. Greater yield response to fungicide was observed at the VT application timing with higher return probability compared to the V6 application (Fig 2). With cost of fungicide application ranging from $30 to $55/ha at a corn price of $0.16/kg, the P*_gain_* ranged from 57 to 27%. The P*_gain_* for the VT application was over 50% when the cost of the fungicide program was below $35/ha at a corn price $0.16/kg.

**Table 5:** Application costs for fungicides ($USD/ha) used in uniform fungicide application timing trials conducted in 2014 and 2015 across the United States and Ontario, Canada.

Product and timing^a^	Mean aerial application cost	Mean ground application cost	Median aerial application cost	Median ground application cost	Number of data points
**Fluxapyroxad + pyraclostrobin, V6**^**b**^	.^c^	$44.35	.	$43.94	17
**Azoxystrobin, V6**	.	$42.22	.	$41.02	14
**Prothioconazole + trifloxystrobin, V6**	.	$31.73	.	$31.20	16
**Picoxystrobin, V6**	.	$28.56	.	$29.62	15
**Fluoxastrobin + flutriafol, V6**	.	$50.47	.	$49.63	12
**Metconazole + pyraclostrobin, VT**^**d**^	$71.21	$64.46	$70.58	$64.63	16
**Prothioconazole + trifloxystrobin, VT**	$53.98	$47.83	$53.18	$45.09	16
**Azoxystrobin + propiconozole, VT**	$54.09	$47.54	$54.98	$47.54	15
**Picoxystrobin, VT**	$50.21	$43.44	$50.40	$43.92	15
**Fluoxastrobin + flutriafol, VT**	$57.46	$50.47	$58.18	$49.63	12
**Cyproconazole + picoxystrobin, VT**	$57.62	$51.31	$57.46	$49.80	13
**Propiconazole, VT**	$23.83	$17.06	$23.90	$15.46	9
**Tetraconazole, VT**	$46.00	$39.02	$44.11	$35.59	12
**Fluxapyroxad + pyraclostrobin, V6 fb**^**e**^ **metconazole + pyraclostrobin, VT**	$122.98	$109.20	$121.92	$110.71	16
**Azoxystrobin, V6 fb azoxystrobin + propiconazole, VT**	$103.80	$89.66	$103.80	$89.66	14
**Prothioconazole + trifloxystrobin, V6 fb prothioconazole + trifloxystrobin, VT**	$92.14	$79.56	$92.62	$79.60	16
**Picoxystrobin, V6 fb cyproconazole + picoxystrobin, VT VT**	$78.65	$64.49	$78.65	$64.49	15
**Fluoxastrobin + flutriafol, V6 fb fluoxastrobin + flutriafol, VT**	$114.89	$100.92	$114.89	$100.92	12

^a^ Rates are listed in Table 1.

^b^ V6 = Six leaf collar corn growth stage.

^c^ “.” Indicates no data available since these applications are typically occur with ground equipment.

^d^ = Tasseling growth stage of corn.

^e^ fb = followed by.

For the DMI + QoI fungicide class, prices ranged from $30 to $80/ha. The probability of offsetting fungicide program cost was greater for DMI + QoI programs compared to the QoI alone program; however, at the V6 application timing, the probability was still not more than 52% in any cost-price combination for DMI + QoI programs. For DMI + QoI fungicides applied at VT, the probability of offsetting fungicide program cost was estimated to be greater than at the V6 application timing, which is consistent with the QoI only products. For DMI + QoI products, the P*_gain_* changed from 73 to 43% when the fungicide program cost increased from $30 to $80/ha at corn price $0.16/kg, while at the V6 application timing the P*_gain_* ranged from 48 to 20% for the same fungicide program cost ($30 to $80/ha) and corn price $0.16/kg. The P*_gain_* was over 50% when the fungicide program cost at VT was below $65/ha at corn price $0.16/kg.

## Discussion

The decision of whether or not to apply a foliar fungicide to hybrid corn has become an annual occurrence in the US and Canada [21]. Farmers and certified crop advisors are most interested in increasing yield and profit in corn production [22], and with tightening profit margins, there is increased farmer interest in establishing the potential for profitability when using fungicides. Our results are consistent with other corn fungicide studies [6, 7], indicating that fungicide application often results in a positive yield response compared with not treating. However, questions remain regarding if yield increases are likely to be profitable, and how application timing influences return on investment. Our analysis demonstrates that certain fungicide classes (QoI, and DMI + QoI) can increase yield and profitability if applied at the VT (tasseling) corn growth stage. The effect size ($\bar{D}$) for V6 (six leaf collar growth stage) applications was positive (127.4 kg/ha) and significantly different from zero, indicating modest yield gains, but the yield response at V6 was less than that for VT (376.8 kg/ha) and V6 + VT applications (493.9 kg/ha). These findings are consistent with previous research indicating that applications occurring at V6 are not likely to result in significant yield increases compared to VT applications [9–15]. In this study, mean yield response observed from VT applications was 2.9 times greater than V6 applications, resulting in a higher probability of return on investment from a fungicide application.

Although yield response to applications occurring at VT and V6 + VT were statistically similar, the probability of recovering costs for programs with two fungicide applications is difficult to achieve with current fungicide program pricing and corn price. Over the two years of this study, application costs across all fungicides for V6 +VT programs ranged from $64.49 to $122.98/ha with an average cost of $88.77/ha for ground application and $102.49/ha for aerial application. This cost is well above the mean ground ($45.14/ha) and aerial ($51.80/ha) application costs for VT applications. Survey results of certified crop advisors and corn growers conducted from 2005 to 2009 reported that only 2.2% of corn farmers were willing to spend $61.78/ha for foliar fungicide application [22], indicating that very few farmers would willingly spend the money required for a V6 + VT application, particularly if yield gains were not different from those observed when applications occur at VT alone.

The marginal yield response observed with V6 fungicide applications has been attributed to the fact that these applications occur too early to reduce foliar disease severity of yield-reducing diseases such as gray leaf spot, northern corn leaf blight, and southern rust. Onset of these diseases typically occur in the later vegetative states (V16) through grain fill, depending on environmental conditions [23]. Few important foliar diseases are present in the early vegetative stages on an annual basis, and currently available foliar fungicides have only 14 to 21 days of residual activity in the plant [8]. Since there are approximately 30 days between growth stage V6 and V16 depending on environmental conditions, little to no fungicide active ingredient will be available in the plant at disease onset, which may explain why V6 applications are less likely to result in higher yield responses and lower probability of economic return. However, in years/locations when disease develops early in the growing season (e.g. during the vegetative phases of corn) in the far southern U.S., greater response to the V6 application may be observed. In this study, the majority of trial locations were in the northern U.S., where authors observe later onset of foliar disease in corn. This might also explain, in part, the nominal response observed at the V6 fungicide application timing. Furthermore, for both QoI and QoI + DMI fungicide classes, power to find differences at the V6 timing was low. This indicates that drawing conclusions about yield differences due to fungicide application at this particular timing, within fungicide class, may require more research. Power was considered adequate or marginally adequate (0.7 for QoI at the VT application timing) for VT and V6+VT application timings.

Despite the link between foliar fungicide yield response in corn and foliar disease severity [3, 7], higher $\bar{D}$ was observed in trials where foliar disease severity was less than 5%. A greater number of trial-studies had high disease severity compared to low disease severity (249 vs. 187), yet $\bar{D}$ for low disease severity was 1.4 times greater than for high disease severity. However, the significance level between $\bar{D}$ for low and high disease severity was marginal with moderate variability about each mean. In addition to the high variability in yield response and disease pressure among trial locations in this study, this result could be due to foliar disease development at later growth stages in some trials. Late-season foliar disease development occurring closer to the rating date (dough through dent (R4-R5)) would have less impact on grain fill and yield response but still result in high foliar disease severity values. Furthermore, other studies that have examined the impact of VT applications of foliar fungicides have observed non-significant effects on yield when significant reductions of foliar disease were observed at greater than 5% severity in the non-treated control [9, 24–27]. Yield increases from VT fungicide applications also have been observed under low disease pressure in other studies [7, 26], and are attributed to the control of lesser foliar diseases and physiological effects caused by fungicides, such as delayed senescence [28]. Yield response when disease severity is low, while documented, is less consistent and ultimately less profitable than when foliar fungicides are used for foliar disease control [3, 7, 26].

Applications of fungicides consisting of a solo QoI or DMI active ingredient are less expensive than products with multiple fungicide classes; however, the probability of a positive return on investment was higher for V6 and VT applications when using DMI + QoI fungicides compared to QoI products alone. There is little published research to support this finding. The majority of previously published multi-location studies focused on the yield effects of QoI fungicides tested alone, or did not separate fungicide products in statistical analyses to determine if class impacts yield response [16, 29, 30]. Additionally, several recent single location studies that compare corn yield response of multiple classes of fungicides have not observed statistical differences in yield responses with QoI + DMI fungicides compared to QoI fungicides alone [29–32], and QoI fungicides are rated as having similar efficacy as QoI + DMI fungicides for important foliar diseases [33]. Therefore, our results may indicate a broader spectrum of disease control or physiological benefits derived from the combination of classes. This finding requires further investigation to determine synergistic effects between fungicide classes [34] and factors influencing this response.

Although survey results of those who recommend and use fungicides demonstrated that foliar fungicides were considered “very” or “extremely” important by 23.9% of respondents [22], the perception of yield gain from fungicide treatment has a significant influence on the decision to apply a fungicide [35, 21]. Of survey respondents indicating foliar fungicide use between 2005 and 2009, 94.4% of CCAs and 65.1% of farmers said they observed a positive yield response in corn [22]. In our research, 68.2% of treatments resulted in a positive yield response from foliar fungicide, which is similar to the perceived response. However, the perceived profitability of yield responses is less well known. Yield responses from foliar fungicides have been well documented, but the probability of a profitable response from fungicide application varied greatly in this study and depended on application timing, fungicide class, application and product cost, and price of corn. Corn prices peaked in the late 2000s, and have decreased in recent years, while fungicide application and product costs have, on average, remained the same. The continued predicted slump in corn pricing indicates that farmers should focus less on yield response alone, and integrate the economic return of yield responses from specific fungicide classes and application timings into the decision-making process.

In addition to economic factors, farmers may want to consider the biological implications of fungicide use. Repeated use of QoI and DMI fungicides has led to fungal pathogen resistance in other cropping systems [3,8], and reduce populations of entomopathogenic fungi, leaving corn more at risk for insect or mite outbreaks [36]. QoI fungicides have also been proven toxic to several aquatic species [37–39]. Additional research is needed to fully understand the impacts of widespread fungicide use on the biological aspects of corn production systems over millions of corn acres in the United States and Canada.

## Supporting information

S1 Fig PRISMA flow diagram. (DOC)

S1 File PRISMA checklist. (DOC)
