# Supplementary material for: Meta-analysis of yield response of foliar fungicide-treated hybrid corn in the United States and Ontario, Canada
Source: PLoS One. 2019 Jun 5;14(6):e0217510. doi: 10.1371/journal.pone.0217510 (PMC6550426; doi:10.1371/journal.pone.0217510)
Supplement: S1 Fig — (DOC) [file pone.0217510.s001.doc]

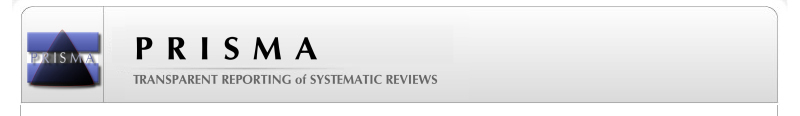
**PRISMA 2009 Flow Diagram**

**Screening**

**Included**

**Eligibility**

**Identification**

Records identified through database searching
(n = N/A)

Additional records identified through other sources
(n = 454 )

Records after duplicates removed
(n = 454 )

Records screened
(n = 454 )

Records excluded
(n = 18 )

Full-text articles assessed for eligibility
(n = N/A )

Full-text articles excluded, with reasons
(n = N/A)

Studies included in qualitative synthesis
(n = 436)

Studies included in quantitative synthesis (meta-analysis)
(n = 436)
